# Validation and Reliability of Polish Version of the Reflux Symptoms Index and Reflux Finding Score

**DOI:** 10.3390/healthcare10081411

**Published:** 2022-07-28

**Authors:** Elżbieta Włodarczyk, Tomasz Jetka, Beata Miaśkiewicz, Piotr Henryk Skarzynski, Henryk Skarzynski

**Affiliations:** 1 Rehabilitation Clinic, World Hearing Center, Institute of Physiology and Pathology of Hearing, 05-830 Warsaw, Poland; 2 World Hearing Center, Institute of Physiology and Pathology of Hearing, 05-830 Warsaw, Poland; t.jetka@gmail.com; 3 Audiology and Phoniatric Department, World Hearing Center, Institute of Physiology and Pathology of Hearing, 05-830 Warsaw, Poland; b.miaskiewicz@ifps.org.pl; 4 Teleaudiology and Screening Department, World Hearing Center, Institute of Physiology and Pathology of Hearing, 05-830 Warsaw, Poland; p.skarzynski@csim.pl; 5 Institute of Sensory Organs, 05-830 Warsaw, Poland; 6 Heart Failure and Cardiac Rehabilitation Department, Faculty of Medicine, Medical University of Warsaw, 03-242 Warsaw, Poland; 7 Otorhinolaryngology Surgery Clinic, World Hearing Center, Institute of Physiology and Pathology of Hearing, 05-830 Warsaw, Poland; h.skarzynski@ifps.org.pl

**Keywords:** validation, reliability, Reflux Finding Score (RFS), Reflux Symptoms Index (RSI), Polish language version

## Abstract

(1) Background: To confirm the credibility, consistency, and replicability of the Polish versions of the Reflux Symptoms Index (PL-RSI) and the Reflux Finding Score (PL-RFS). (2) Methods: The translation followed the WHO recommendations. The study group included 100 volunteers (age 15–87) with hoarseness and pharyngolaryngeal complaints. The control group comprised 55 healthy volunteers (age 20–75). Study participants completed the PL-RSI; then, two independent otolaryngologists completed the PL-RFS based on pharyngeal videostroboscopy. Questionnaires were repeated after 7 days, with no treatment before the second round. Additionally, patients underwent 24 h pH-metry. The control group had a single round of questionnaires followed by pH-metry. (3) Results: The PL-RSI is consistent, reliable (Cronbach’s alpha 0.77–0.83; test–retest reliability 0.83), and significantly correlated with other patient-filled tools (*p* < 0.001). The PL-RFS intra-rater reliability is 0.84–0.91, and inter-rater is 0.88. Both questionnaires strongly correlate with pH-metry (PL-RSI upright Ryan Score 0.35, PL-RFS—0.60). Both clearly distinguish (i) healthy from persons with voice disorders, but without acid LPR (*p* < 0.0001), and (ii) within patient group between subjects with and without acid LPR (*p* = 0.0002). (4) Conclusions: The PL-RSI and PL-RFS are reliable and can be recommended to Polish-speaking otolaryngologists. Our findings confirm the role of country-specific factors in RSI results and that practitioners should always use a proper control group.

## 1. Introduction

The Reflux Symptoms Index (RSI) and the Reflux Finding Score (RFS) are two questionnaires proposed by Belafsky [1,2] for measuring the intensity of laryngopharyngeal reflux (LPR)-related symptoms; they are designed to make LPR diagnosis easier and more accessible. The Reflux Symptoms Index (RSI) measures a patient’s subjective perception of their daily level of illness. The Reflux Finding Score (RFS) is completed by a specialist based on laryngoscopy or videostroboscopy.

Their disadvantage is that symptom severity is assessed either subjectively by the patient (in the case of RSI) or by a specialist performing the laryngoscopy (for RFS). Thus, questionnaire results may be significantly affected by the demographics and culture of a specific population, the way in which the medical terms used in the questionnaires are understood, and other linguistic and translation issues.

Polish versions of these two questionnaires lack a reliable analysis of their consistency and efficacy [3]. Our preliminary studies conducted on adults [3,4] as well as children and teenagers [5] show that at least one of these problems typically occurs. Thus, to ensure diagnostic reliability and usability of the questionnaires, formal validation of the translations is required, followed by a study with a reference group, as suggested by the questionnaires’ authors [1,2,6]. Such formal validation has been performed in several countries, including the USA, France, Turkey, the Philippines, and Korea [7,8,9,10,11,12,13,14,15,16,17,18,19]. The observed differences are significant [13], meaning that adaptation of the tools to local conditions is essential. At the same time, even though RSI and RFS are commonly accepted and used, their effectiveness in confirming the presence of LPR is still in doubt, and studies on this subject are contradictory [15,20,21,22,23,24,25,26,27]. Even with the original, English-language versions of these questionnaires, there are doubts as to the way they are used [28,29] and whether a correct diagnosis is possible based only on a description of symptoms (even though it is agreed that the questionnaires are useful for measuring the effects of treatment) [30]. The original study performed by Belafsky confirmed that these two questionnaires were excellent tools for measuring the effects of treatment of LPR and proving the efficacy of the chosen therapy. Other studies have confirmed their usefulness [20,31,32,33]. However, Belafsky has also provided reference values that allow one to confirm the presence of LPR based on the 95% confidence level of two control groups (Belafsky used just 40 persons for the RFS scale and 25 for the RSI scale). Although the author advises caution because “findings consistent with reflux are present in asymptomatic individuals without a clinical diagnosis of LPR” [1], an RSI score higher than 13 or an RFS higher than 7 have been accepted as indicators of LPR and are commonly used in its diagnosis [7,32].

There are several reasons why using the RSI and RFS questionnaires for diagnosis may be problematic. First, the RSI questionnaire measures the patient’s subjective perception of symptoms. The problem here may arise from a country’s specific culture or translation of the questionnaire [7,29], or other psychological aspects [31]. Second, questionnaires are usually targeted to particular populations of patients, which may differ depending on the aim of the study [34]. For example, it is hard to determine the overlap of the reference values between one country and another. Some studies also indicate problems with the repeatability of RFS depending on the diagnostician [35]. All these considerations make it difficult to fully transfer the original recommendations and apply them to Poland, and they may lead to misleading outcomes. Indeed, such factors may underlie why there are reports in the literature where RSI and RFS results are inconsistent with the observed LPR. In the case of RFS, reports often indicate that a relationship is lacking [36,37], while with RSI, the results are sometimes inconsistent [36,37,38]. Published reports from different countries confirm that the above factors may significantly affect the reliability of RSI and RFS. Reports on the German [30] and Greek [8] language versions confirm their diagnostic usability, but reports on the Korean [36] and Indian [29] versions point to multiple practical problems. The Italian [7] and Arabic [9] translations are considered reliable, but creation of these versions was preceded by intensive analysis and followed by systematic translation and validation.

Therefore, having in mind the practical use of the RSI and RFS questionnaires in Poland, we conducted the following study with the objective to ensure that our instruments represent reliability, consistency, and replicability for the target population and as such are justified diagnostic tools.

## 2. Materials and Methods

### 2.1. RSI and RFS Translation

The translation of both questionnaires followed a similar procedure to arrive at PL-RSI and PL-RFS. We adopted the recommendations of the World Health Organization [39,40,41] for the translation and cross-cultural adaptation of instruments in health care. To begin, an initial translation of the American version of each of the tests [1,2] was prepared by a multidisciplinary team composed of two general otolaryngologists, a senior otolaryngology professor, two expert speech therapist, one epidemiologist, one linguist, and one statistician, all native speakers of Polish, together with a native English speaker. Then, this initial version was sent out to a panel of experts who had practical experience in administering diagnostics tests for voice disorders throughout Poland. After receiving feedback, a draft PL-RSI was shown to nonmedical personnel at our clinic, asking them about ambiguous or too specialized language. From the comments received, two intermediate versions of the PL-RSI test and one intermediate version of the PL-RFS were developed until a consensus opinion was reached within our committee. Finally, the accepted version was sent to a bilingual English–Polish speaker for back-translation. As it happened, no additional changes were required. Finally, using PL-RSI, a pre-test cognitive interview was conducted with 25 patients to verify its comprehensibility.

### 2.2. Participants

We enrolled 100 volunteers in the study from all patients who visited the Clinic of Audiology and Phoniatrics between January 2017 and June 2019 due to chronic or temporary hoarseness accompanied by other pharyngeal and laryngeal complaints. The youngest was 15 years old and the oldest was 87.

In addition, 55 volunteers were enlisted as a control group from healthy relatives and friends of other patients who attended the Clinic between June 2018 and June 2019. We ensured that the main study group and the control group were independent, were not related, and did not overlap. The volunteers from the control group ranged from 19 to 72 years old.

A subject was not accepted into the study or control group if one of the following conditions was met: they had previously been diagnosed with asthma, head and neck or digestive tract malignancy, history of ear, nose, and throat radiotherapy, seasonal allergy, untreated thyroid disease, prior anti-reflux surgery, alcohol or tobacco abuse, or suffered from other significant medical or surgical disease. Additionally, those being treated for a peptic ulcer (with antisecretory or anti-Helicobacter pylori therapy) or using acetylsalicylic acid or other nonsteroid anti-inflammatory drugs (NSAIDs) were excluded.

### 2.3. RSI and RFS Procedure

All participants in the main study group were asked to complete the PL-RSI scale on the day of admission to the study. Next, the pharynx was assessed using videolaryngostroboscopy (VLS), after which the PL-RFS questionnaire was filled in separately by two independent, experienced otolaryngologists. After 7 days, the two questionnaires were filled in once more, and the VLS was repeated (with the PL-RSI being filled in by the patient and the PL-RFS by the two experts). To ensure the two sets were comparable, participants were asked not to undergo any treatment before the second round of tests was performed. To prevent memory bias, neither the patient nor the interviewer had access to the initial PL-RSI questionnaire and neither did the physicians have access to the previous VLS or PL-RFS results.

None of the participants dropped out of the study between day 1 and day 7.

Independently, the healthy volunteers were examined in a single round of filling in the PL-RSI and PL-RFS questionnaires, followed by pH-metry.

### 2.4. pH-Metry and LPR Diagnosis

Together with the initial examination by the PL-RSI and PL-RFS scales, patients were asked to participate in a 24 h pH-metric procedure.

A Restech^®^ pH sensor was calibrated in solutions of pH 7.0 and pH 4.0 before use. A catheter was inserted transnasally and advanced until the flashing LED was seen at the back of the subject’s throat; it was then positioned so that the flashing light was 5–10 mm below the uvula. The diameter of the LED light was 5 mm, and it served as a placement guide. The probe was secured to the patient’s face, as close to the nares as possible, using a Tegaderm™, and then passed over the ear and secured to the neck with a second Tegaderm™. The probe’s transmitter was taped to the skin or attached to the subject’s clothing with a clip. A data recorder was attached to the patient’s belt. Patients were asked not to shower during the recording period and to keep a diary indicating meal times and the time spent horizontal (in bed) and vertical (out of bed). Meals were excluded from the analysis of pharyngeal pH recordings (extending them by 5 min on either side). Data from the Restech^®^ recorder were downloaded to a proprietary software program and correlated with the patient’s diary. The analysis involved counting the number of times (number of reflux episodes) that the pH dropped below 5.5 when upright or below 5.0 when horizontal, as well as the duration of these events (expressed as a percentage of the total monitoring time).

Acid LPR was diagnosed based on the Ryan score, which was calculated as the percentage of time that the pharyngeal pH was below the above-mentioned thresholds, the number of episodes during which the pH dropped, and the duration of the drops (separately for vertical and horizontal body positions). According to the manufacturer, a Ryan score above 9.41 for the upright position and above 6.8 for the horizontal is considered abnormal [28,38].

### 2.5. Other Measurement and Diagnostic Tools

In addition, the VHI scale by Jacobson [42] and the Warsaw Scale by Domeracka-Kołodziej [43] were used at the baseline of the study. To test the basic internal consistency, we employed an internal questionnaire with seven questions about the strength and frequency of symptoms, physical well-being, mood, social contact, everyday functioning, and general health. Participants were asked to answer each question using a numerical scale between 1 (low, bad, rarely) and 5 (high, good, often). Non-sensitive demographic variables, including sex, age, weight, and others were collected as auxiliary data.

### 2.6. Reliability Analysis

For reliability analysis of both PL-RSI and PL-RFS we looked at both internal consistency and temporal stability. In addition, for the PL-RFS scale, we evaluated intraobserver reliability.

*Internal consistency* refers to the degree to which each question of a scale relates to all the other questions. The most widely used method to measure this parameter is Cronbach alpha (α). A high coefficient suggests the items within a dimension measure the same construct, supporting the construct’s validity. A value greater than 0.7 is considered “satisfactory”, greater than 0.8 “good”, and greater than 0.9 “excellent.” To prevent memory bias, Cronbach α was measured only for the initial round of PL-RSI.

*Temporal stability* is also known as test–retest reliability. It refers to the concordance between the scores of repeated measurements from the same participant. It can be assessed by the intraclass correlation coefficient (ICC). A coefficient over 0.80 is considered a high value. The test−retest reliability was measured by comparing the initial and repeated responses for all individual items as well as the total score of the PL-RSI and PL-RFS scales.

*Intra-observer reliability* for the RFS scale was calculated by applying the ICC between the scores given by the independent experts.

### 2.7. Validity Assessment

A comprehensive validity procedure was conducted, which included three main elements:

1. Comparison with the control group. The rationale behind conducting PL-RSI and RFS in the healthy subjects was two-fold: (i) it allowed us to establish a country-specific baseline and reference group; (ii) the difference between the group with voice disorders and a group without symptoms shows the relevance of the scale to the problem it was meant to solve.
2. Concordance with other LPR diagnostic tools. One of the aims was to determine the extent that measures from other LPR scales relate to PL-RSI and PL-RFS.
3. Relationship to pH-metry measurements. The gold standard of acid LPR detection is assumed to be pharyngeal pH-metry measurements. Hence, the basal validity and usability of PL-RSI and PL-RFS was evaluated by their predictive power for diagnosis of acid LPR and by the correlation between the value of pH-metry and the strength of symptoms as indicated by the scales.

### 2.8. Statistical Method

All comparisons of quantitative variables between groups, e.g., the construct validity between study and control groups, were assessed using a two-sample Student’s *t*-test. To measure dependence, the Spearman coefficient was calculated, while its significance was tested with Algorithm AS 89 as implemented in the R statistical package. The independence between two categorical variables was assessed by Fisher’s exact test.

For a better presentation of Ryan scores, both horizontal and upright, we calculated the logarithm of the Ryan score, logRyan, according to the formula:

logRyan = ln(Ryan + 1)

where Ryan is the Ryan score in either the horizontal or upright position.

Computations were carried out in R. In hypothesis testing, it was assumed that a *p*-value below 0.05 indicated rejection of the null hypothesis. All figures were prepared with R’s ggplot2 package. In the box plots here, boxes indicate the 1st and 3rd quartiles, the horizontal line represents the median, and the vertical line represents the range between the lowest and highest value (unless the value exceeded the 1st or 3rd quartiles by more than 1.5 interquartile ranges). Cronbach α and the ICCs were obtained by functions implemented in the *psych* R package.

### 2.9. Ethics Statement

The study was performed following the ethical standards laid down in the Declaration of Helsinki. The study protocol was approved by the Research and Ethics Committee of the Institute of Physiology and Pathology of Hearing at the World Hearing Center, Poland (IFPS:KB/11/2017). All participants were invited to volunteer and after acceptance signed a formal consent form and were enrolled in the study. Permission to translate the original RSI and RFS into PL-RSI and PL-RFS was obtained from Peter C. Belafsky (University of California, Davis, CA, USA).

## 3. Results

### 3.1. Translation and Validation Procedure

Table 1 lists the items of the PL-RSI after the formal and iterated translation. For details of how this was performed, see the Methods section.

Table 2 sets out the RFS scale translated into Polish in a similar way, although it did receive more feedback from practitioners following daily use. Although the country-specific differences are more evident for the RSI scale, we were still surprised that even a trained otolaryngologist was unsure about the true meaning of each of the items. We also found relatively high levels of uncertainty among physicians about whether each RFS item should allow for a zero score or not. A practical outcome from our study was obtaining general agreement from Polish specialists in voice disorders to use the version of the scale presented in Table 2 and which is now distributed by our clinic.

### 3.2. Study Group: PL-RSI and PL-RFS Scores

In the main study group, we examined 100 subjects (65 women and 45 men) with an average age of 49.55 years (SD = 13.84).

Table 3 presents total scores for PL-RSI and PL-RFS for each of the two rounds of examination (test and re-test). In the case of RFS, each test result from the two independent experts is listed, and is complemented by basic demographics, VHI, Ryan score, and LPR status.

### 3.3. Consistency and Reliability of PL-RFS

The developed PL-RSI is a consistent reliable scale.

Table 4 shows that the overall consistency of the PL-RSI is good, and justified by Cronbach α of 0.77 and 0.83 for the test and re-test scenarios, respectively. Additionally, excluding any of the items from the scale did not change the consistency significantly. Moreover, the correlation of each item with the total RSI score was high and ranged between 0.48 and 0.79, demonstrating that all items are important for assessing symptoms. The re-test reliability of 0.83 (0.7–0.86 for separate items) is also acceptable. Together, these statistics suggest that the Polish translation of the RSI questionnaire is consistent and reliable.

### 3.4. Consistency and Reliability of PL-RFS

The PL-RFS scale is reliable within and between independent experts.

The overall picture of PL-RFS reliability in our population is somewhat more complicated than for PL-RSI. Firstly, Item 7 of the PL-RFS was almost never detected—it was noted twice by expert 1, and once by expert 2. Therefore, the calculation of reliability statistics was not possible for Item 7 due to the sample size being too small.

Table 5 reports two metrics: intra-rater reliability (test–retest reliability between the same expert) and inter-rater reliability (correlation between expert 1 and expert 2)—both calculated using ICC.

The overall intra-rater reliability was satisfactory—expert 1 had an ICC of 0.91, while expert 2 achieved an ICC of 0.84. Almost all items of PL-RFS had an intra-rater ICC greater than 0.8. Two exceptions were Item 2 for expert 1 (ICC = 0.76) and Item 8 for expert 2 (ICC = 0.69). Nonetheless, all these reliability statistics achieved high statistical significance (*p* < 1 × 10^−10^). The inter-rater reliability was also high (ICC = 0.88). The highest discrepancy between the experts corresponded to Item 1 of PL-RFS, where the ICC was 0.57.

### 3.5. Validity of PL-RFS and PL-RSI

Validity analysis was carried out in 3 steps: correlation with other LPR diagnostic tools, correlation with pH-metry read-outs, and differences.

PL-RSI is significantly associated with other patient-filled tools.

Table 6 shows that the correlation between the PL-RFS and PL-RSI scales was low (*r* = 0.15) and insignificant (*p* = 0.14). Nonetheless, total PL-RSI corresponded quite well to other symptom measurements tools. Thus, correlation with the VHI scale ranged from 0.34 to 0.39 (*p* < 0.001), while correlation with the simple 5-level symptom perception scale was 0.54 for symptom strength (*p* < 0.001) and 0.33 (*p* = 0.001) for symptom frequency. On the other hand, RFS was insignificantly associated with VHI scales (*r* = 0.09–0.16, *p* > 0.1) and just significant for symptom perception by patients (symptom strength, *r* = 0.2, *p* = 0.05; symptom frequency, *r* = 0.21, *p* = 0.03).

**Figure 1:**
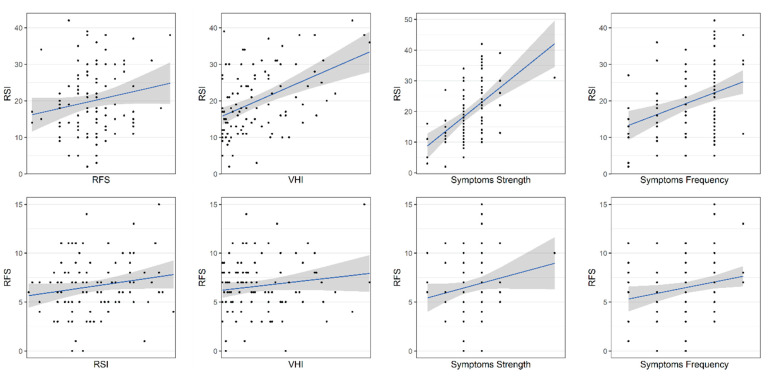
Quantitative dependence between PL-RSI, PL-RFS, and other LPR diagnostic tools shown as scatter plots and fitted linear regression lines. Grey areas indicate 95% confidence intervals.

Figure 1 shows scatter plots of the calculated measures of dependence and linear regression lines. Interestingly, a weak relationship between PL-RSI and PL-RFS was also observed universally at the level of individual items.

**Figure 2:**
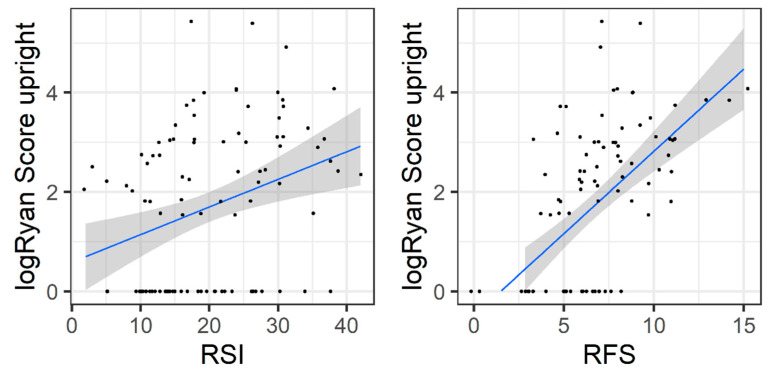
PL-RSI and PL-RFS scales against logRyan score measured in the upright position. The blue line is the fitted linear regression, while the grey area is the 95% confidence interval.

Next, we evaluated the prognostic power of PL-RFS and PL-RSI tools as assessed by comparison with objective diagnostic pH-metry measurements. As measured with pH monitoring, there was a clear correlation between reflux presence and PL-RFS and PL-RSI scores. The PL-RSI had a correlation of 0.35 (*p* < 0.001) with upright logRyan score and–0.02 (*p* = 0.86) with supine logRyan score. The adequacy of PL-RFS was even higher and was strongly associated with higher reflux presence both for upright and supine: the correlation of PL-RFS with supine logRyan score was 0.31 (*p* = 0.002) and with upright it was 0.6 (*p* < 0.001). The data are shown as scatter plots in Figure 2.

**Figure 3:**
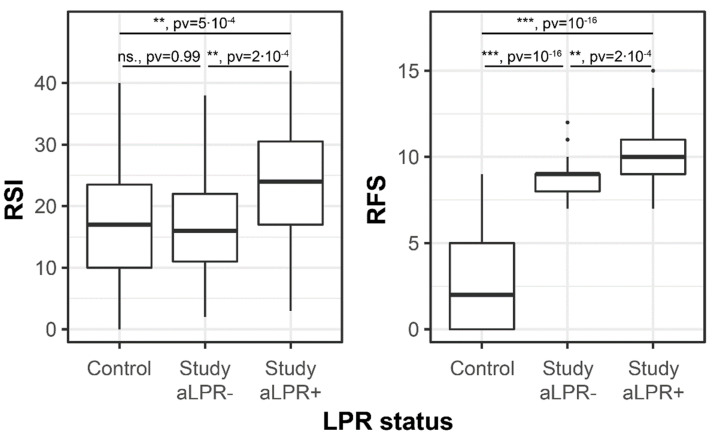
Comparison of PL-RSI and PL-RFS between those in the study group who were identified with acid LPR (aLPR+), those who were acid LPR negative (aLPR-), and the healthy controls (Control). Significance of Student’s *t*-test was: ** *p* < 0.01, *** *p* < 10^−6^.

Patients were divided into two groups according to pH monitoring results: severe acid LPR positive (aLPR+) and severe acid LPR negative (aLPR-), and Figure 3 shows there was a significant difference in PL-RSI and PL-RFS between those two groups. For the aLPR+, the mean total PL-RSI score was 23.9, and for the aLPR- group, the mean was 17.1; a Student’s *t*-test indicated a statistical difference (*p* < 0.001) and an effect size of 0.75. Similarly, aLPR+ patients scored an average of 10 on PL-RFS, whereas aLPR- patients scored 8.8. A Student’s *t*-test for the difference in PL-RFS between those two groups showed an effect size of 0.76 (*p* < 0.001). In general, these results indicate that PL-RSI and PL-RFS are both valid scales to diagnose LPR problems.

PL-RSI and PL-RFS clearly discriminate between the acid LPR and control groups. Our control group of 55 patients did not have any known or reported voice disorders. We presented them with the PL-RSI questionnaire, conducted a videostroboscopy examination, and evaluated them with PL-RFS. The findings for PL-RSI and PL-RFS were different, as shown in Figure 3. There was no statistical difference between PL-RSI conducted in the control group and those patients from the study group who did not show the LPR phenotype in pH-metry (*p* = 0.99). On the other hand, patients with diagnosed acid LPR had significantly higher PL-RSI scores than those without severe acid LPR diagnosis (mean 23.9 vs. 17.1, *p* = 0.0005). The PL-RFS scale provided a higher level of differentiation, and both comparisons proved to be significant: participants of the main study group without acid LPR had a higher mean RFS than the control group (8.79 vs. 2.76, *p* < 10^−16^) and subjects with acid LPR had significantly elevated PL-RFS versus those without LPR (mean of 9.95 vs. 8.79, *p* = 0.0002).

Nonetheless, it must be noted that the observed mean value of PL-RSI in the reference group is quite high (17.1), resulting in a lower overall sensitivity for PL-RSI. In comparison to the standards proposed by Belfasky, i.e., a cut-off value of 13, almost 60% of healthy subjects in our control group scored above this threshold.

## 4. Discussion

The RSI and RFS questionnaires are recognized and used worldwide in the diagnosis and therapy of LPR. Whereas the RFS is filled in by a specialist, the RSI questionnaire is filled in by the patient, and this raises doubts, notwithstanding that “patient-centered medicine”, focusing not so much on the disease as the affected patient, is playing an increasingly important role. The use of a self-administered questionnaire allows one to gauge the effectiveness of a treatment from the patient’s point of view. Importantly, a questionnaire allows data to be acquired in a quick, low-cost way. As with any other tool used in diagnosis, a questionnaire needs to be reliable, give a reliable measurement, and be a valid measure of the examined phenomenon. Here, the aim of adapting and validating the two questionnaires was to establish the credibility of the Polish-language RSI and RFS versions so that they might be effectively used in clinical practice.

This is the first validated Polish version of RSI and RFS. The results of our own study demonstrate that the consistency and reliability of the measurements using the PL-RSI and PL-RFS questionnaires were high. Our adaptation of the RSI questionnaire to the Polish language involved a rigorous and iterated process of translation and revision. The resulting PL-RSI has been proven to be a consistent and reliable questionnaire. At the same time, we have confirmed that the PL-RFS scale is also a reliable tool within and between independent experts. Importantly, we found that PL-RSI correlated significantly with other patient-filled tools.

Schindler et al. [7] validated the Italian version of RSI. Its psychometric properties were studied in 80 patients with a RFS (>7), and 193 asymptomatic subjects were included in the study as a control group. The results showed strong internal consistency (*α* = 0.99), high test–retest reliability (*r* > 0.90), and optimal clinical validity. However, the authors suggested that age-related differences could be an important factor when evaluating a suspected LPR. The comparison between the RSI scores of the pathological and the control groups was statistically significant, allowing symptomatic subjects to be distinguished from asymptomatic ones. In the study, the correlation between RFS score and RSI score was high (*r* = 0.89). Moreover, although a correlation between each item of the RSI and the RFS total score was demonstrated, the authors considered that no single RSI symptom could be used as a definite marker for LPR. We agree with the author that, in order to increase the probability of detection, both questionnaires should be used together in clinical practice.

Lechien et al. [12] developed a French version of the Reflux Symptom Index (Fr-RSI) and assessed its internal consistency, reliability, and clinical validity in a group of 44 patients aged 23–84 years old with RFS (>7) and RSI (>13). Additionally, included in the study were 90 asymptomatic people (aged 19–60 years). Validity was assessed by comparing Fr-RSI scores with scores from the Voice VHI in 24 of the 44 patients. The test–retest reliability was high in patients with LPR (*r*_BP_  =  0.78) and in healthy subjects (*r*_BP_  =  0.80). Cronbach alpha was 0.85, indicating high internal consistency. Based on an item discrimination analysis, the authors suggested that throat clearing, troublesome cough, globus sensation, and heartburn were the symptoms most correlated with LPR disease. In practical terms, Fr-RSI together with RFS (with appropriate thresholds RSI  >  13 and RFS  >  7) can be widely used among the French-speaking population.

Akbulut et al. [13] validated and assessed the reliability of the Turkish RSI in a group of 132 patients (aged 16–71 years old) with a RFS >7, comparing them to 162 healthy controls with RFS ≤ 7. The T-RSI was scored twice within a 7–14 day window. The T-RSI showed excellent internal consistency with a Cronbach α of 0.912. Furthermore, Cronbach α obtained when any T-RSI item was deleted demonstrated that all the items were consistent with the questionnaire, and deletion of any item decreased the level of internal consistency. Test–retest reliability measured using Pearson’s correlation coefficient was 0.93 for total T-RSI, supporting that it has high stability and reproducibility. The T-RSI demonstrated clinical validity, with a significant difference between the RSI scores of the study and control groups. A strength of the study was its large sample size and the sensitivity and specificity of the T-RSI was determined using an ROC curve. The authors said that due to difficulties associated with ambulatory pH monitoring they preferred to differentiate patients from control participants with RFS using fiberoptic laryngoscopy. There was a high correlation between the RSI total score and RFS in 132 patients in the study. They concluded that scores greater than 12.5 are suggestive of LPR and that T-RSI can be used together with RFS in routine evaluation and clinical management.

In the study by Calvo-Henriquez et al. [14], the authors asked patients to fill out a Spanish version of the RSI twice—the first time during an examination in the office, and a second time by telephone. There were 150 people (aged 13–97) with and without symptoms of laryngopharyngeal reflux who participated in the study. The authors did not change the original version of the questionnaire, and Cronbach α was high in the general population (0.872) as well as in the gender and age subgroups. Interestingly, consistency was slightly higher in the male population (0.897 for males, 0.852 for females), which the authors explained by the large number of women in the study who were menopausal and had nonspecific throat symptoms.

Eckley and Tangerina [19] studied the psychometric properties of the Brazilian Portuguese version of the RSI in a group of 154 subjects: 88 patients with LPR and 66 controls. The authors considered that patients who had objective proof of reflux ascending into the upper esophagus and pharynx (positive prolonged double-probe pH-metry or MII-pH tests), and patients with chronic laryngitis of no other probable cause were found to have erosive esophagitis on EGD as representative of reflux-related chronic laryngitis. They conclude that when attempting to diagnose LPR, one must be very careful to rule out other possible causes of chronic irritation of the larynx and pharynx. When there is a strong clinical suspicion of LPR, the RSI has proven to be a helpful diagnostic and prognostic tool.

Farahat et al. [9] developed an Arabic version of the RSI in a group of 52 patients with suspected LPR and in 100 control subjects. The overall estimated internal consistency of the Arabic RSI was satisfactory for the study group (α = 0.72). The results of the Arabic RSI showed a statistically significant difference between the patients and the control groups, for both the overall and the individual item scores. When the total scores of Arabic RSI were compared with the total scores of Arabic VHI-10, there was no significant correlation. The authors explained that patients with LPR will feel affected (handicapped) if they are asked about the effect of LPR on their voice. Moreover, they suggested that Arab people put more emphasis on the emotional impact of their voice problems relating to LPR than the physical and functional effects.

In our work, we used 24 h pharyngeal pH measurements to confirm the occurrence of acid LPR. Previously, this method has not been used to validate the RSI and RFS questionnaires. Although this method does seem to be a good diagnostic tool, it has its limitations, as other authors have indicated.

For example, Lechien et al. [12] reported that the technique can lead to false-negative or false-positive diagnoses due to variations in probe placement or movement of the probe during monitoring. According to Jacob et al. [42] and Sataloff et al. [44], false-positive rates range from 7 to 17%. According to Calvo-Henríquez et al. [14], pH-metry tests have certain flaws in diagnosing LPR and cannot be performed on all patients suspected of presenting LPR, especially given their invasiveness and high cost. In the case of the tests carried out here, the same doctor placed the probe according to strict rules so as to minimize false-positives and false-negatives caused by different placement of the probe. Akbulut et al. [13] considered that pH monitoring was time-consuming, relatively invasive, expensive, and not tolerated by all patients. Certainly, in routine clinical practice, invasive monitoring cannot be used on all patients, although the patients participating in this study tolerated it well. pH monitoring is a test that even children can tolerate, but we acknowledge that the technique is expensive. Salivary pepsin tests, although much cheaper and invasive, are still to be validated and have not become standard in clinical practice.

The relevance of both questionnaires has been demonstrated by their strong association with pH-metry measurements. From a practical perspective, PL-RSI and PL-RFS can together be used to distinguish each of the following groups: healthy subjects (H), acid LPR-negative patients with voice disorders (aLPR–), and severe acid LPR patients (aLPR+). Previous research has proven that RSI has good psychometric properties, which allows it to be reliably used in clinical practice.

We believe that these versions of the RSI and RFS questionnaires validated in Polish have good clinical relevance and can be used in clinical practice for the diagnosis of LPR. Like other authors in other languages, we are of the opinion that the use of both scales at the same time significantly improves their diagnostic value.

## 5. Conclusions

In this study, we successfully carried out formal translation of the RSI questionnaires for diagnosing LPR in Polish-speaking patients. In parallel work, we also clarified concerns about the use of the RFS questionnaire in the Polish language. Subsequently, we have demonstrated that both Polish questionnaires are characterized by:

- High reliability and consistency;
- Correlation with the objective clinical metrics of acid LPR;
- Adequate distinction between patients with and without severe acid LPR.

In summary, we recommend the use of PL-RSI and PL-RFS to all Polish-speaking otolaryngologists. At the same time, our findings strengthen the idea that there are substantial country-specific factors at play when using RSI and there is a need for practitioners to use a proper reference control group.

## Figures and Tables

**Table 1:** Final version of the translated PL-RSI questionnaire.

Item Number	Item in English RSI	Item in Polish PL-RSI	Scale
1	Hoarseness or a problem with your voice	Chrypka lub problemy głosowe	0–5
2	Clearing your throat	Chrząkanie	0–5
3	Excess throat mucus or postnasal drip	Nadmierna ilość wydzieliny w gardle lub spływania wydzieliny po tylnej ścianie gardła	0–5
4	Difficulty swallowing food, liquids, or pills	Zaburzenia połykania	0–5
5	Coughing after you eat or after lying down	Kaszel po jedzeniu lub położeniu się	0–5
6	Breathing difficulties or choking episodes	Zaburzenia oddychania	0–5
7	Troublesome or annoying cough	Męczący kaszel	0–5
8	Sensation of something sticking to your throat or a lump in your throat	Uczucie przeszkody lub ciasnoty w gardle	0–5
9	Heartburn, chest pain, indigestion, or stomach acid coming up	Pieczenie/ból w klatce piersiowej, niestrawność, uczucie cofania się treści żołądkowej	0–5
	Total RSI		0–45

**Table 2:** The RFS scale with each item translated into Polish.

Item Number	Item in English RFS	Item in Polish RFS	Scores
1	Pseudosulcus (infraglottic edema)	Obrzęk okolicy podgłośniowej	0, 2
2	Ventricular obliteration	Obliteracja kieszonki krtaniowej	0, 2, 4
3	Erythema and hyperemia	Rumień/przekrwienie	0, 2, 4
4	Vocal fold edema	Obrzęk fałdów głosowych	0–4
5	Diffuse laryngeal edema	Rozległy obrzęk krtani	0–4
6	Posterior commissure hypertrophy	Przerost spoidła tylnego	0–4
7	Granuloma/granulation tissue	Ziarniniak/ziarnina	0, 2
8	Thick endolaryngeal mucus	Gęsty wydzielina śluzowa w okolicy zanalewkowej i/lub zachyłków gruszkowatych	0, 2
	Total RSI		0–26

**Table 3:** Baseline statistics of the main study group.

		All	Female	Male
N		100 (100%)	65 (65%)	35 (35%)
Age		Mean: 49.55 (SD: 13.84)	Mean: 49.23 (SD: 13.14)	Mean: 50.14 (SD: 15.22)
RSI	TEST	Mean: 20.2 (SD: 8.5)	Mean: 20.55 (SD: 8.41)	Mean: 19.54 (SD: 8.75)
	RE-TEST	Mean: 20.02 (SD: 9.13)	Mean: 20.66 (SD: 9.04)	Mean: 18.83 (SD: 9.3)
RFS (L1)	TEST	Mean: 6.62 (SD: 2.8)	Mean: 6.66 (SD: 2.53)	Mean: 6.54 (SD: 3.28)
	RE-TEST	Mean: 6.7 (SD: 3)	Mean: 6.8 (SD: 2.82)	Mean: 6.51 (SD: 3.33)
RFS (L2)	TEST	Mean: 6.51 (SD: 2.82)	Mean: 6.52 (SD: 2.78)	Mean: 6.49 (SD: 2.93)
	RE-TEST	Mean: 6.62 (SD: 2.67)	Mean: 6.65 (SD: 2.62)	Mean: 6.57 (SD: 2.8)
VHI	overall	Mean: 20.49 (SD: 20.29)	Mean: 22.48 (SD: 21.71)	Mean: 16.8 (SD: 17.04)
	functional	Mean: 5.28 (SD: 6.65)	Mean: 5.55 (SD: 7.15)	Mean: 4.77 (SD: 5.67)
	emotional	Mean: 9.65 (SD: 9.4)	Mean: 10.92 (SD: 9.83)	Mean: 7.29 (SD: 8.17)
	physical	Mean: 5.56 (SD: 6.35)	Mean: 6 (SD: 6.79)	Mean: 4.74 (SD: 5.42)
Symptom perception	strength	Mean: 3.37 (SD: 0.98)	Mean: 3.35 (SD: 1.01)	Mean: 3.4 (SD: 0.95)
	frequency	Mean: 3.26 (SD: 1.08)	Mean: 3.15 (SD: 1.09)	Mean: 3.46 (SD: 1.04)
Ryan score	upright	Mean: 16.9 (SD: 35.58)	Mean: 21.48 (SD: 42.69)	Mean: 8.39 (SD: 11.93)
	supine	Mean: 1.21 (SD: 4.84)	Mean: 1.08 (SD: 3.98)	Mean: 1.43 (SD: 6.19)
LPR status	LPR	43	30	13
	no LPR	57	35	22

**Table 4:** Reliability of the PL-RSI scale.

RSI Item	Cronbach Alpha (if Item Deleted)		Correlation with Total RSI		Test–Retest Reliability
	TEST	RE-TEST	TEST	RE-TEST	
Item 1	0.76 (SE: 0.037)	0.83 (SE: 0.026)	0.50	0.58	0.80
Item 2	0.75 (SE: 0.037)	0.82 (SE: 0.026)	0.52	0.58	0.74
Item 3	0.75 (SE: 0.037)	0.83 (SE: 0.026)	0.56	0.61	0.80
Item 4	0.75 (SE: 0.038)	0.82 (SE: 0.027)	0.53	0.61	0.86
Item 5	0.73 (SE: 0.04)	0.8 (SE: 0.031)	0.65	0.79	0.70
Item 6	0.73 (SE: 0.04)	0.81 (SE: 0.028)	0.57	0.62	0.79
Item 7	0.72 (SE: 0.042)	0.81 (SE: 0.028)	0.71	0.69	0.85
Item 8	0.75 (SE: 0.038)	0.82 (SE: 0.027)	0.60	0.63	0.73
Item 9	0.76 (SE: 0.036)	0.82 (SE: 0.027)	0.48	0.61	0.74
	**Cronbach’s alpha**				**Test–retest reliability**
	**TEST**		**RE-TEST**		
**Total RSI**	0.77 (95% CI: 0.7–0.84)		0.83 (95% CI: 0.79–0.88)		0.83

Correlation between an item and the total score was computed as Spearman correlation coefficients. Test–retest reliability was computed using interclass correlation coefficients (ICC). SE indicates standard error; 95% CI is 95% confidence interval.

**Table 5:** Reliability of the PL-RFS scale.

RFS Item	Intra-Rater Reliability				Inter-Rater Reliability	
	Expert 1		Expert 2			
	ICC	*p*-Value	ICC	*p*-Value	ICC	*p*-Value
Item 1	1.00	0.00 × 10^0^	0.84	1.50 × 10^−18^	0.57	1.93 × 10^−5^
Item 2	0.76	6.10 × 10^−20^	0.81	1.80 × 10^−16^	0.66	1.00 × 10^−7^
Item 3	0.83	1.80 × 10^−26^	0.85	5.70 × 10^−19^	0.78	5.78 × 10^−13^
Item 4	0.89	8.00 × 10^−35^	0.80	9.20 × 10^−16^	0.84	2.40 × 10^−18^
Item 5	0.89	1.30 × 10^−35^	0.84	2.50 × 10^−18^	0.83	1.03 × 10^−16^
Item 6	0.92	3.10 × 10^−42^	0.86	2.80 × 10^−20^	0.83	3.51 × 10^−17^
Item 7	NA	NA	NA	NA	NA	NA
Item 8	0.83	3.50 × 10^−26^	0.69	8.30 × 10^−11^	0.84	1.11 × 10^−17^
RFS Total	0.91	1.38 × 10^−38^	0.84	8.52 × 10^−19^	0.88	9.47 × 10^−23^

**Table 6:** Correlation between PL-RSI and PL-RFS scales and the other instruments evaluated.

		PL-RSI		PL-RFS	
		*r*	*p*-Value	*r*	*p*-Value
**PL-RSI**		1.00	0.00	0.15	0.14
PL-RFS		0.15	0.14	1.00	0.00
VHI	overall	0.40	4.00 × 10^−5^	0.11	0.26
	functional	0.34	5.80 × 10^−4^	0.16	0.11
	physical	0.39	6.60 × 10^−5^	0.13	0.19
	emotional	0.36	2.30 × 10^−4^	0.09	0.39
Symptom perception	strength	0.54	8.40 × 10^−9^	0.20	0.05
	frequency	0.33	8.80 × 10^−4^	0.21	0.03

*r*—Spearman correlation coefficient.

## Data Availability

Not applicable.
